# Nocturnal fat oxidation is lower in older individuals with overweight/obesity, including those with type 2 diabetes, and is associated with fasting triglyceride levels

**DOI:** 10.1007/s00125-026-06736-z

**Published:** 2026-05-12

**Authors:** Soraya S. de Kam, Jeroen H. P. M. van der Velde, Anna Veelen, Johanna A. Jörgensen, Joris Hoeks, Patrick Schrauwen

**Affiliations:** 1 Department of Nutrition and Movement Sciences, Maastricht University Medical Center+, Maastricht, the Netherlands; 2 Department of Clinical Epidemiology, Leiden University Medical Center, Leiden, the Netherlands; 3 Institute for Clinical Diabetology, German Diabetes Center, Leibniz Institute for Diabetes Research at Heinrich Heine University Düsseldorf, Düsseldorf, Germany; 4 German Center for Diabetes Research (DZD), München Neuherberg, Germany

**Keywords:** Energy expenditure, Indirect calorimetry, Metabolic flexibility, Nocturnal fat oxidation, Obesity, Substrate metabolism, Type 2 diabetes

## Abstract

**Aims/hypothesis:**

We previously showed that nocturnal fat oxidation is reduced in older individuals with overweight/obesity and impaired glucose tolerance and insulin sensitivity compared with young lean individuals. Here, we compared nocturnal energy expenditure and substrate oxidation across groups varying in age, body composition and metabolic status to unravel factors underlying variations in nocturnal substrate metabolism.

**Methods:**

Data were collected from 18 previously conducted human clinical studies (*N*=187), all performed under conditions of energy balance with similar diet composition and meal timing. Individuals were categorised into four groups: young lean (YL); older lean (OL); older with overweight/obesity (OBE); and older with overweight/obesity and type 2 diabetes (T2D). Nocturnal energy expenditure and substrate oxidation were determined by whole-room indirect calorimetry, body composition was assessed by air-displacement plethysmography or dual energy x-ray absorptiometry, and glucose, insulin, HOMA-IR, NEFA and triglycerides were measured from fasted blood samples. Group comparisons for nocturnal energy expenditure and substrate oxidation were performed using Kruskal–Wallis tests, and over time using linear mixed models including group × time interactions, with Bonferroni correction applied to both analyses. Multivariate linear regression analysis was applied to identify whether age, sex, HOMA-IR, fasting NEFA, fasting triglycerides, fat mass and fat-free mass were independent factors of nocturnal energy expenditure and substrate oxidation.

**Results:**

Nocturnal energy expenditure, adjusted for fat-free mass, was higher in OBE compared with YL and OL (*p*<0.01 for both); it was also higher in T2D compared with OL (*p*<0.01). Nocturnal fat oxidation, expressed as a percentage of energy expenditure, was lower in OBE (median: 46.28%, IQR: 37.74–53.05) and T2D (median: 46.48%, IQR: 41.05–53.65) compared with YL (median: 52.95%, IQR: 47.82–57.61; *p*<0.01 for both) and OL (median: 55.21%, IQR: 54.15–58.89; *p*<0.01 for both). Standardised linear regression models revealed that fasting triglycerides were positively associated with nocturnal respiratory exchange ratio (β=0.337; 95% CI 0.165, 0.508) and nocturnal carbohydrate oxidation (% of energy expenditure; β=0.337; 95% CI 0.166, 0.509), and inversely associated with nocturnal fat oxidation (% of energy expenditure; β=−0.352; 95% CI −0.520, −0.813).

**Conclusions/interpretation:**

Nocturnal fat oxidation (% of energy expenditure) is diminished in older individuals with overweight/obesity, irrespective of diabetes status. No differences in nocturnal energy expenditure (adjusted for fat-free mass) or substrate oxidation were observed between young and older lean individuals, suggesting that age per se may not strongly influence nocturnal substrate metabolism. Fasting triglyceride level was the strongest associated factor of nocturnal substrate oxidation.

**Graphical Abstract:**

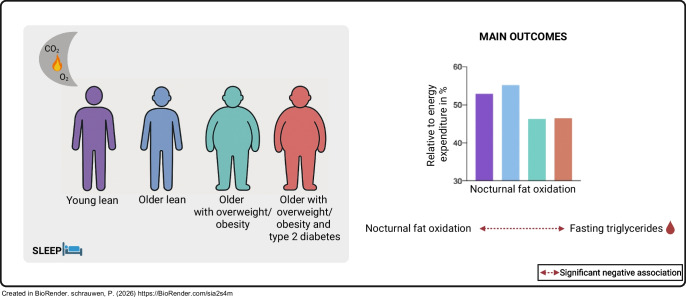

**Supplementary Information:**

The online version contains peer-reviewed but unedited supplementary material available at 10.1007/s00125-026-06736-z.


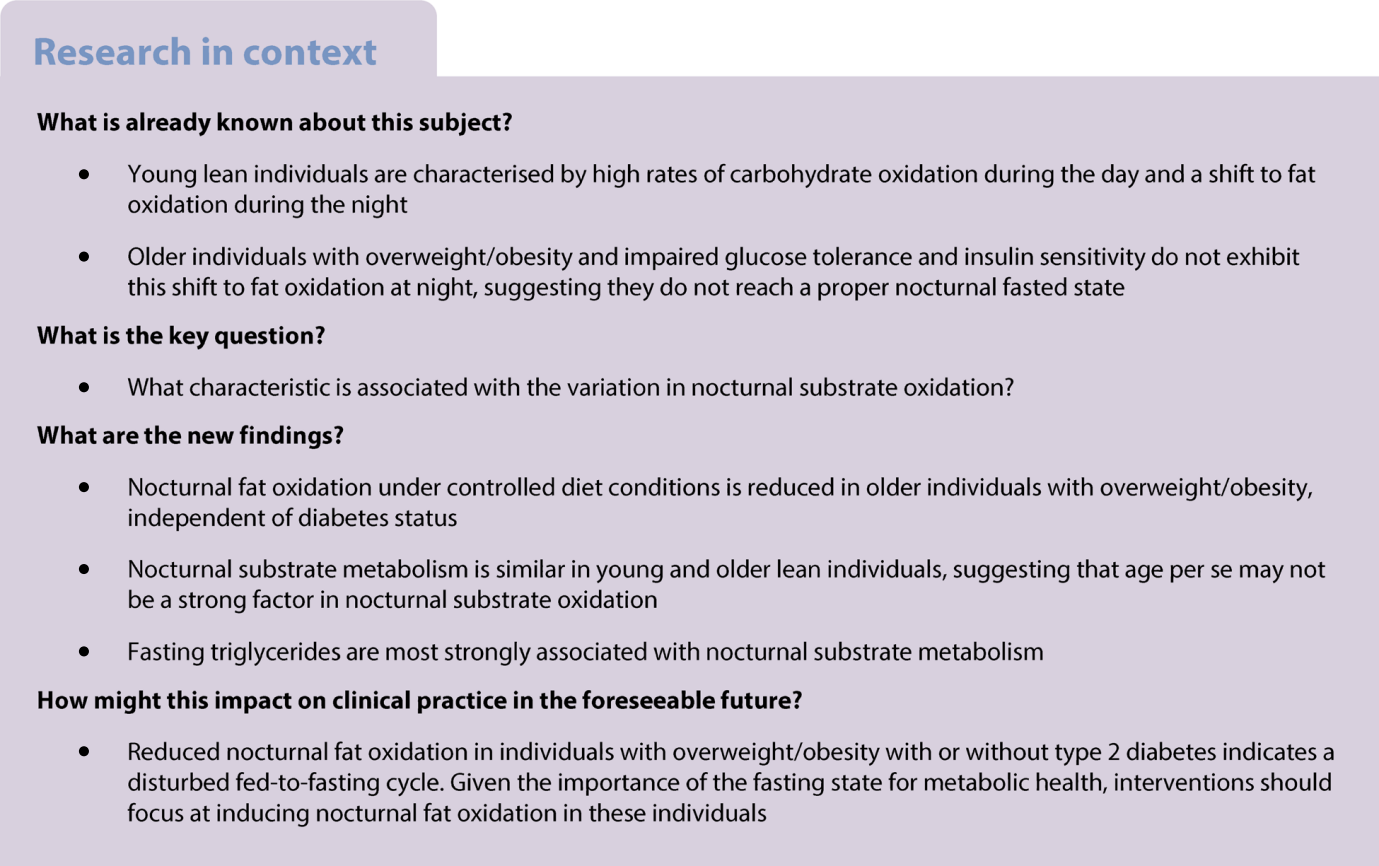


## Introduction

Metabolic inflexibility, the inability to rapidly adjust substrate utilisation to substrate availability and requirement, is a contributor to metabolic disorders such as type 2 diabetes [1–3]. In healthy conditions, elevated glucose availability stimulates insulin release by pancreatic beta cells, which promotes glucose uptake in skeletal muscle, liver and adipose tissue while simultaneously inhibiting lipolysis in adipose tissue, thereby favouring glucose oxidation. Conversely, in the absence of exogenous glucose or during fasting, insulin levels drop while glucagon levels rise, stimulating lipolysis in adipose tissue and promoting a shift towards higher fat oxidation.

We previously demonstrated a 24 h rhythmicity in energy expenditure and substrate oxidation, consistent with feeding and fasting periods, in young lean individuals. Specifically, a high carbohydrate oxidation and a low fat oxidation was demonstrated in the fed state, with a robust change towards lower carbohydrate oxidation and a higher fat oxidation in the (overnight) fasted state [4]. Interestingly, we subsequently found that such fed-to-fasting transition in substrate utilisation was absent in older individuals with overweight/obesity and impaired glucose tolerance and insulin sensitivity. Compared with young lean individuals, these individuals exhibit lower nocturnal fat oxidation and higher nocturnal carbohydrate oxidation, indicating a reduced capacity to switch to fat oxidation at night, and consequently do not reach a true nocturnal fasted state, despite having similar and controlled (timing of) food intake and physical activity [5].

The exact mechanisms underlying such metabolic inflexibility are unknown. Greater body fat has been associated with elevated postprandial glucose, while higher BMI has been associated with increased postprandial triglycerides (TG) following a standardised liquid meal challenge [6]. These unfavourable glycaemic and lipid responses suggest that body composition may influence metabolic flexibility. Metabolic inflexibility may also be associated with metabolic status, particularly through dysregulated plasma metabolites. Older individuals with overweight/obesity and impaired glucose tolerance and insulin sensitivity exhibit higher daytime levels of postprandial glucose, insulin and TG along with lower levels of NEFA compared with young lean individuals. These differences persist at night, with elevated TG and suppressed NEFA, even when food intake is controlled [5]. It is also well known that 24 h rhythmicity in metabolism is influenced by the biological clock, which is blunted in obesity and insulin resistance but also in ageing [5, 7–9].

Here, we compared nocturnal energy expenditure and substrate oxidation in a variety of populations: young lean individuals (YL); older lean individuals (OL); older individuals with overweight/obesity (OBE); and older individuals with overweight/obesity and type 2 diabetes (T2D), to identify which characteristics are associated with the variation in nocturnal substrate oxidation between these groups. To this end, data from a total of 18 human clinical studies with comparable study design were included in the present study.

## Methods

### Study design

Data were combined from 18 previously performed human clinical trials (*N*=187) conducted at Maastricht University, the Netherlands, all performed under standardised conditions (electronic supplementary material [ESM] Table 1). All clinical trials were approved by the Ethics Committee of Maastricht University Medical Center and registered at ClinicalTrials.gov or trialregister.nl, and completed between 2011 and 2025. Participants provided written informed consent for the original studies and studies were performed in accordance with the Declaration of Helsinki. All clinical trials recruited participants via advertisement from the vicinity of Maastricht. The included participants comprised both young and older adults, with a predominance of men. No data on ethnicity or socioeconomic factors were available. Participants from the combined clinical trials were divided into four groups differing in age, body composition and metabolic status: 37 YL (age: 18–30 years, BMI <25 kg/m^2^); ten OL (age: 40–75, BMI <25 kg/m^2^); 92 OBE (age: 40–75, BMI ≥25 kg/m^2^); and 48 T2D (age: 40–75, BMI ≥25 kg/m^2^; ESM Fig. 1). Participants in the T2D group had been diagnosed with type 2 diabetes for at least 6 months and were well-controlled on glucose-lowering medication (e.g. metformin, sulfonylurea agents and/or dipeptidyl peptidase-4 [DPP-4] inhibitors) or were drug naive for at least 3 months prior to the study. Depending on the set-up of the clinical trial, data were obtained from either the screening or measurements before or at the end of the placebo-controlled or control condition. If participants enrolled in more than one of these studies, only data from the (first) study with the most complete set of measurements were included.

### Indirect calorimetry

Oxygen consumption (${\dot{V}\mathrm{O}}_{2}$) and carbon dioxide production (${\dot{V}\mathrm{CO}}_{2}$) at night were continuously measured using whole-chamber indirect calorimetry (Omnical, Maastricht Instruments, Maastricht, the Netherlands) [10]. From these measurements, energy expenditure (EE), carbohydrate oxidation and fat oxidation were calculated using the Brouwer equation [11]. Protein oxidation was estimated to contribute 12.4% of total EE [12]. Respiratory exchange ratio (RER) was determined by the ratio of ${\dot{V}\mathrm{CO}}_{2}$ to ${\dot{V}\mathrm{O}}_{2}$. Calculations for EE, RER and substrate oxidation were performed over a 4.5 h period during the night (00:30 to 05:00 hours), and during three distinct periods (00:30 to 02:00 hours, 02:00 to 03:30 hours and 03:30 to 05:00 hours) to analyse changes in pattern substrate metabolism over time.

### Blood sampling and analysis: fasting glucose, insulin, NEFA and TG

Fasted blood samples were obtained via an intravenous cannula and collected in serum or EDTA-containing tubes and centrifuged at 1300 *g* for 10 min at room temperature for serum and at 4°C for EDTA plasma. Aliquots were frozen in liquid nitrogen and stored at −80°C for subsequent analysis. Glucose (Horiba, Montpellier, France) and NEFA (WAKO Chemicals, Neuss, Germany) were quantified calorimetrically using a Cobas Pentra C400 analyser (Horiba, Montpellier, France). TG (Sigma-Aldrich, St Louis, Missouri, USA; Roche Diagnostics, Mannheim, Germany; or DiaSys Diagnostic Systems, Holzheim, Germany) levels were quantified calorimetrically using a Cobas Pentra C400 analyser or the KoneLab clinical analyser (Thermo Fisher Scientific, Vantaa, Finland). Insulin was measured using an ELISA kit (Crystal Chem, Elk Grove Village, Illinois, USA) or an autoanalyser (ARCHITECT ci16200 analyser; Abbott Laboratories, Abbott Park, Illinois, USA).

#### Body composition

Fat mass (FM) and fat-free mass (FFM) were determined using air-displacement plethysmography (BodPod; Cosmed, Rome, Italy) or dual energy x-ray absorptiometry (DEXA; Discovery A, Hologic Corp, Bedford, Massachusetts, USA), depending on the set-up of the clinical trial.

#### Statistics

Nocturnal EE was adjusted for the main determinant, FFM, and expressed as unstandardised residuals, to which the overall mean EE (across all groups) was added. Data from participants who did not undergo BodPod or DEXA assessments were excluded (*n*=27) from the calculation of nocturnal FFM-adjusted EE (ESM Fig. 1).

Statistical analyses were conducted using SPSS Statistics version 28.0.1.0 (IBM, Armonk, New York, USA) and GraphPad Prism version 10.6.1 (GraphPad Software, San Diego, California, USA). Results were presented as median values with IQR. The Kruskal–Wallis test was used to compare nocturnal (00:30 to 05:00 hours) EE, RER, carbohydrate oxidation and fat oxidation between groups, followed by Dunn’s post hoc test with Bonferroni correction for multiple comparisons. A *p* value of <0.05 was considered statistically significant.

To determine whether nocturnal EE, RER, carbohydrate oxidation and fat oxidation changed over time and whether these changes differed between groups, a linear mixed model (LMM) was conducted, with the night divided into three sections (00:30 to 02:00 hours, 02:00 to 03:30 hours, 03:30 to 05:00 hours). The best fitting covariate structure was selected based on the lowest Akaike’s Information Criterion (AIC). If statistical differences were observed, pairwise comparison was performed with Bonferroni correction for multiple comparisons. A *p* value of <0.05 was considered statistically significant.

Finally, a multiple linear regression analysis was performed to identify which characteristics were most strongly associated with nocturnal EE and substrate oxidation. All included variables had a variance inflation factor (VIF) <5 and tolerance >0.2, indicating no multicollinearity among variables. Following crude analysis, the associations were additionally adjusted for age, sex, HOMA-IR, fasting NEFA, fasting TG, FM, and FFM, with results expressed as regression coefficients and 95% CI. Individuals with incomplete data were excluded (*n*=42) to avoid bias and ensure consistency across all regression analyses. Furthermore, the YL group was excluded from this analysis since the sample size was low after excluding individuals with incomplete data on variables, and most differences would be driven by age (ESM Fig. 1). In addition, to compare the strength of predictors for nocturnal EE and substrate oxidation, a standardised (*z* score) regression analysis was performed, with results expressed as standardised regression coefficients (β) and 95% CI. A regression coefficient was considered statistically significant if its 95% CI did not include zero.

## Results

### Characteristics

Participants were divided into groups differing in age, body composition and metabolic status and comprising 37 YL, ten OL, 92 OBE and 48 T2D participants, with participant characteristics listed in Table 1. By design, YL was younger than OL, and body mass and FM were higher in OBE and T2D compared with YL and OL groups. Fasting glucose and TG levels were lowest in the YL group and highest in the T2D group. Fasting insulin levels were lowest in the OL group, with similar values in YL, OBE and T2D groups. Fasting NEFA and HOMA-IR were lowest in the OL group and highest in the T2D group.

**Table 1:** Characteristics between study groups: YL, OL, OBE and T2D

Characteristic	YL	OL	OBE	T2D
Sex, men (%) *n*	100 37	70 10	73 92	65 48
Age, years *n*	22 (21–24) 37	67 (64–69) 10	66 (59–70) 92	67 (62–70) 48
BMI, kg/m^2^ *n*	22.4 (21.4–24.4) 37	24.0 (22.0–25.5) 10	30.1 (28.3–32.6) 92	28.9 (27.4–31.2) 48
Fasting glucose, mmol/l *n*	5.1 (4.9–5.3) 36	5.8 (5.4–6.0) 10	5.5 (5.2–6.0) 92	8.0 (7.4–9.1) 47
Fasting insulin, pmol/l *n*	68.8 (41.4–88.0) 36	23.6 (13.2–44.4) 10	64.6 (41.6–102.6) 92	65.5 (48.0–87.0) 46
Fasting TG, mmol/l *n*	0.8 (0.7–1.1) 37	1.5 (1.1–1.8) 10	1.7 (1.2–2.4) 91	1.8 (1.3–2.2) 47
Fasting NEFA, mmol/l *n*	0.5 (0.3–0.7) 37	0.4 (0.4–0.5) 10	0.5 (0.3–0.7) 82	0.6 (0.4–0.8) 46
Body mass, kg *n*	74.0 (67.3–77.0) 11	69.4 (61.2–80.5) 10	91.2 (81.1–100.0) 92	85.6 (80.0–96.8) 47
FM, kg *n*	14.4 (11.7–20.1) 11	20.1 (17.4–22.2) 10	32.8 (27.5–36.7) 92	30.5 (26.2–34.9) 47
FFM, kg *n*	56.4 (54.7–58.1) 11	53.8 (37.5–60.5) 10	57.9 (49.6–65.9) 92	58.2 (49.5–64.3) 47
HOMA-IR *n*	2.3 (1.3–3.0) 36	0.8 (0.5–1.5) 10	2.5 (1.4–3.4) 92	3.4 (2.5–4.7) 46

Data are presented as median (25th−75th percentile)

Group size: YL: *n*=37; OL: *n*=10; OBE: *n*=92; and T2D: *n*=48. Sample sizes for individual variables may differ due to missing data

### Nocturnal whole-body EE, adjusted for FFM, is elevated in individuals with overweight/obesity with or without type 2 diabetes

**Fig. 1:**
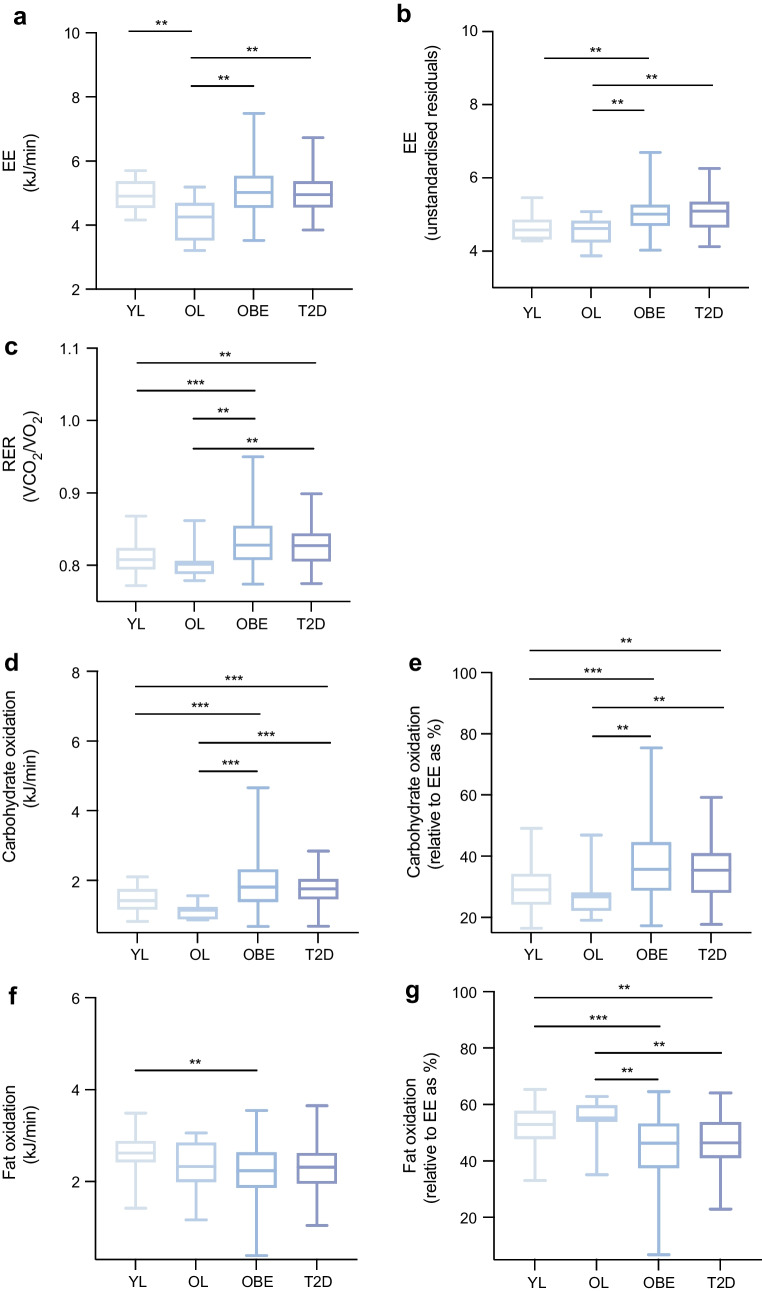
Nocturnal EE, RER and substrate oxidation across YL, OL, OBE and T2D groups. (**a**) Nocturnal EE (kJ/min); (**b**) nocturnal EE (adjusted for FFM, kg); (**c**) nocturnal RER; (**d**) nocturnal carbohydrate oxidation (kJ/min); (**e**) nocturnal carbohydrate oxidation (relative to EE as %); (**f**) nocturnal fat oxidation (kJ/min); and (**g**) nocturnal fat oxidation (relative to EE as %). Data presented in the boxplots display the median and range. Group size: YL: *n*=37; OL: *n*=10; OBE: *n*=92; and T2D: *n*=48. Sample sizes for panel (**b**): YL: *n*=11; OL: *n*=10; OBE: *n*=92; and T2D: *n*=47, due to missing body composition data. Differences are considered significant at *p*<0.05. **p<0.01; ***p<0.001 based on Bonferroni post hoc test

Absolute nocturnal EE (kJ/min) differed across groups (*p*=0.008), with higher values in YL, OBE and T2D compared with OL (*p*=0.004, *p*=0.002 and *p*=0.002, respectively), while no differences were observed between YL, OBE and T2D (Fig. 1a). When adjusted for FFM (*p*=0.001), nocturnal EE was higher in OBE and T2D compared with OL (*p*=0.002 and *p*=0.004, respectively) and in OBE compared with YL (*p*=0.006). No differences were observed between the other groups (Fig. 1b, ESM Table 2).

**Fig. 2:**
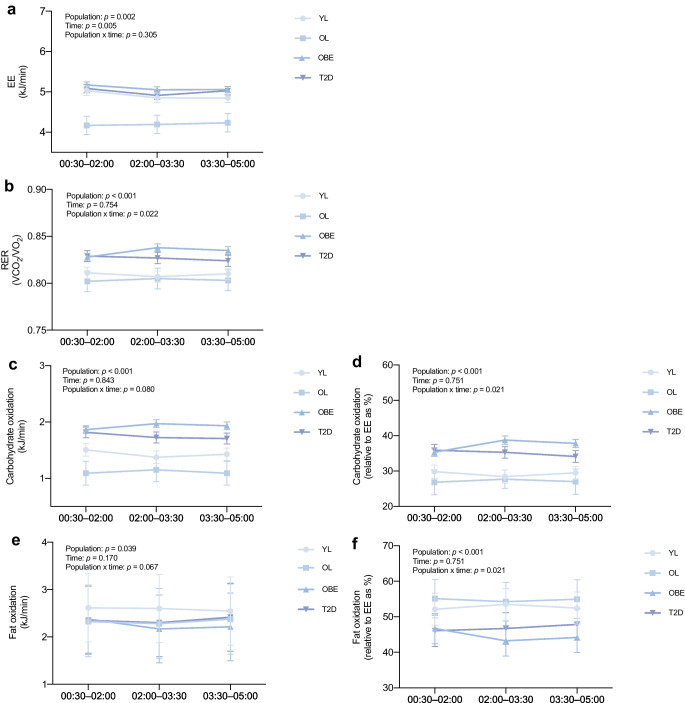
Nocturnal EE, RER and substrate oxidation over the course of the night across YL, OL, OBE and T2D groups. (**a**) Nocturnal EE (kJ/min); (**b**) nocturnal RER; (**c**) nocturnal carbohydrate oxidation (kJ/min); (**d**) nocturnal carbohydrate oxidation (relative to EE as %); (**e**) nocturnal fat oxidation (kJ/min); and (**f**) nocturnal fat oxidation (relative to EE as %). Data are presented as mean with SEM. Group size: YL: *n*=37; OL: *n*=10; OBE: *n*=92; and T2D: *n*=48. Differences are considered significant at *p*<0.05

Since EE typically declines across the night, we explored whether this temporal change differed between groups by dividing the night into three parts: 00:30 to 02:00 hours, 02:00 to 03:30 hours and 03:30 to 05:00 hours. A main effect of time was observed for absolute nocturnal EE (*p*=0.005), with higher nocturnal EE during the first part of the night compared with the middle part (mean: 4.86, SEM: 0.07 vs mean: 4.75, SEM: 0.07; *p*=0.004; Fig. 2a). No population × time interaction was found (ESM Table 3).

### Nocturnal whole-body substrate oxidation is blunted in individuals with overweight/obesity with or without type 2 diabetes

Nocturnal RER differed between groups (*p*<0.001), with higher values in OBE (median: 0.83, IQR: 0.81–0.85) and T2D (median: 0.83, IQR: 0.81–0.84) compared with YL (median: 0.81, IQR: 0.79–0.82; *p*<0.001 and *p*=0.001, respectively) and OL (median: 0.80, IQR: 0.79–0.81; *p*=0.004 and *p*=0.006, respectively), but with no differences between YL and OL or OBE and T2D (Fig. 1c). In line with this, absolute nocturnal carbohydrate and fat oxidation (kJ/min) also differed between groups (*p*<0.001 and *p*=0.014, respectively; Fig. 1d and f, respectively). Absolute nocturnal carbohydrate oxidation was higher in OBE (median: 1.81 kJ/min, IQR: 1.40–2.30) and T2D (median: 1.76 kJ/min, IQR: 1.47–2.01) compared with YL (median: 1.42 kJ/min, IQR: 1.18–1.73) and OL (median: 1.15, kJ/min IQR: 0.88–1.20; *p*<0.001, *p*=0.001, *p*<0.001 and *p*<0.001, respectively), whereas absolute nocturnal fat oxidation was significantly lower in OBE (median: 2.23 kJ/min, IQR: 1.87–2.63) compared with YL (median: 2.62 kJ/min, IQR: 2.42–2.88; *p*=0.002). No other group differences were observed. Since absolute nocturnal EE differed between groups, nocturnal carbohydrate and fat oxidation were also expressed relative to EE (%). Similar results were found for nocturnal carbohydrate oxidation when expressed relative to EE (Fig. 1e), whereas nocturnal fat oxidation relative to EE (Fig. 1g) was lower in OBE (median: 46.28%, IQR: 37.74–53.05) and T2D (median: 46.48%, IQR: 41.05–53.65) compared with YL (median: 52.95%, IQR: 47.82–57.61; *p*<0.001 and *p*=0.001, respectively) and OL (median: 55.21%, IQR: 54.15–58.89; *p*=0.004 and *p*=0.006, respectively). No other group differences were observed (ESM Table 2).

### Individuals with overweight/obesity shift towards carbohydrate oxidation later in the night

Since it is known that substrate oxidation can also change during the night, shifting towards more (fasting-induced) fat oxidation towards the end of the night, we also explored whether nocturnal substrate oxidation differentially changed over time between groups. Although no main effect of time was found for nocturnal RER, populations showed different patterns across the night (population × time: *p*=0.022; Fig. 2b). Nocturnal RER remained stable in YL, OL and T2D, whereas OBE had lower RER in the first part of the night compared with the middle and last parts (*p*<0.001 and *p*=0.038, respectively). No time or interaction effects were found for absolute nocturnal carbohydrate or fat oxidation (Fig. 2c and e, respectively). When relative to EE, nocturnal carbohydrate and fat oxidation showed no time effect, but populations exhibited different patterns across the night (population × time=0.021 for both; Fig. 2d and f, respectively). In OBE, carbohydrate oxidation was lower and fat oxidation was higher in the first part of the night compared with the middle (*p*<0.001 for both) and last parts (*p*=0.035 for both), while they remained stable in the other groups (ESM Table 3).

### Associations of age, sex, HOMA-IR, fasting NEFA, fasting TG, FM and FFM with nocturnal EE and substrate oxidation

We next looked for factors of nocturnal EE and substrate oxidation by performing a linear regression analysis and displaying models adjusted for various covariates: associations in model 1 were adjusted for age and sex; associations in model 2 were additionally adjusted for FM and FFM; and associations in model 3 were additionally adjusted for HOMA-IR, fasting NEFA and TG.

As presented in Table 2, no significant associations were found between age, sex, HOMA-IR or fasting NEFA and nocturnal EE or substrate oxidation after adjustment for covariates (model 3). By contrast, higher fasting TG levels (per 1 mmol/l) were associated with 0.013 (95% CI 0.006, 0.019) higher nocturnal RER (model 3), 0.209 (95% CI 0.089, 0.329) kJ/min higher absolute nocturnal carbohydrate oxidation (model 3) and 4.288% (95% CI 2.171, 6.405) higher nocturnal carbohydrate oxidation relative to EE (model 3). Furthermore, higher fasting TG level was associated with 0.220 (95% CI −0.337, −0.103) kJ/min lower absolute nocturnal fat oxidation (model 3) and 4.245% (95% CI −6.400, −2.090) lower nocturnal fat oxidation relative to EE (model 3). No significant associations were found between fasting TG and absolute nocturnal EE after adjustments for covariates (model 3). Following adjustments for covariates, greater FM (per 1 kg) was associated with 0.024 (95% CI 0.016, 0.033) kJ/min higher absolute nocturnal EE (model 3) and 0.014 (95% CI 0.002, 0.025) kJ/min higher absolute nocturnal fat oxidation (model 3) but not with RER, carbohydrate oxidation (absolute and relative to EE) or fat oxidation relative to EE. Higher FFM (per 1 kg) was associated with 0.050 (95% CI 0.039, 0.061) kJ/min greater absolute nocturnal EE (model 3) and 0.029 (95% CI 0.014, 0.045) kJ/min greater absolute nocturnal carbohydrate oxidation (model 3), but not with RER, carbohydrate oxidation relative to EE or fat oxidation (absolute and relative to EE) when adjusted for covariates.

**Table 2:** Linear regression analyses examining associations between participant characteristics and nocturnal EE and substrate oxidation

Variable	EE (kJ/min)^a^	RER^a^	Carbohydrate oxidation (kJ/min)^a^	Carbohydrate oxidation (%)^a^	Fat oxidation (kJ/min)^a^	Fat oxidation (%)^a^
Age, years						
Model 1^b^	−0.022 (−0.035, −0.009)	−0.001 (−0.001, 0.000)	−0.022 (−0.036, −0.008)	−0.243 (−0.479, −0.006)	0.004 (−0.009, 0.017)	0.241 (0.006, 0.476)
Model 2^c^	0.001 (−0.009, 0.010)	−0.000 (−0.001, 0.000)	−0.011 (−0.025, 0.004)	−0.180 (−0.441, 0.080)	0.011 (−0.003, 0.025)	0.179 (−0.080, 0.438)
Model 3^d^	0.000 (−0.010, 0.009)	0.000 (−0.001, 0.000)	−0.007 (−0.021, 0.007)	−0.107 (−0.360, 0.145)	0.007 (−0.006, 0.021)	0.107 (−0.144, 0.358)
Sex, men vs women						
Model 1^b^	0.900 (0.675, 1.125)	0.010 (−0.002, 0.023)	0.477 (0.240, 0.714)	3.436 (−0.649, 7.520)	0.262 (0.037, 0.488)	−3.416 (−7.476, 0.645)
Model 2^c^	0.197 (−0.058, 0.453)	0.004 (−0.017, 0.025)	0.080 (−0.309, 0.468)	1.397 (−5.683, 8.477)	0.082 (−0.297, 0.462)	−1.389 (−8.428, 5.649)
Model 3^d^	0.191 (−0.072, 0.454)	−0.004 (−0.025, 0.016)	−0.064 (−0.447, 0.318)	−1.465 (−8.368, 5.439)	0.220 (−0.153, 0.593)	1.456 (−5.407, 8.319)
HOMA-IR						
Model 1^b^	0.046 (0.005, 0.088)	0.001 (−0.001, 0.003)	0.028 (−0.016, 0.072)	0.352 (−0.409, 1.114)	0.010 (−0.032, 0.052)	−0.350 (−1.107, 0.407)
Model 2^c^	0.003 (−0.026, 0.032)	0.001 (−0.002, 0.003)	0.010 (−0.034, 0.054)	0.267 (−0.535, 1.068)	−0.008 (−0.051, 0.035)	−0.265 (−1.062, 0.532)
Model 3^d^	0.002 (−0.027, 0.032)	0.000 (−0.002, 0.002)	−0.005 (−0.048, 0.038)	−0.033 (−0.809, 0.743)	0.007 (−0.035, 0.049)	0.033 (−0.739, 0.804)
Fasting NEFA, mmol/l						
Model 1^b^	0.258 (−0.216, 0.732)	0.011 (−0.014, 0.037)	0.281 (−0.218, 0.781)	3.686 (−4.933, 12.305)	−0.069 (−0.546, 0.408)	−3.664 (−12.233, 4.904)
Model 2^c^	0.222 (−0.099, 0.543)	0.012(−0.015, 0.039)	0.305 (−0.183, 0.794)	3.888 (−5.042, 12.818)	−0.122 (−0.601, 0.358)	−3.865 (−12.743, 5.012)
Model 3^d^	0.223 (−0.102, 0.548)	0.010 (−0.016, 0.035)	0.273 (−0.199, 0.745)	3.174 (−5.345, 11.693)	−0.089 (−0.549, 0.371)	−3.156 (−11.625, 5.313)
Fasting TG, mmol/l						
Model 1^b^	0.010 (−0.112, 0.133)	0.013 (0.006, 0.019)	0.213 (0.089, 0.337)	4.263 (2.166, 6.360)	−0.203 (−0.321, −0.085)	−4.238 (−6.323, −2.154)
Model 2^c^	−0.011 (−0.092, 0.070)	0.013 (0.006, 0.019)	0.210 (0.092, 0.327)	4.288 (2.171, 6.405)	−0.217 (−0.332, −0.103)	−4.263 (−6.368, −2.158)
Model 3^d^	−0.015 (−0.097, 0.068)	0.013 (0.006, 0.019)	0.209 (0.089, 0.329)	4.288 (2.171, 6.405)	−0.220 (−0.337, −0.103)	−4.245 (−6.400, −2.090)
FM, kg						
Model 1^b^	0.035 (0.026, 0.045)	0.000 (0.000, 0.001)	0.015 (0.003, 0.026)	0.088 (−0.114, 0.290)	0.014 (0.003, 0.025)	−0.087 (−0.289, 0.114)
Model 2^c^	0.026 (0.018, 0.033)	0.000 (0.000, 0.001)	0.010 (−0.002, 0.021)	0.061 (−0.149, 0.272)	0.011 (0.000, 0.022)	−0.061 (−0.270, 0.148)
Model 3^d^	0.024 (0.016, 0.033)	−1.792 × 10^−5^ (−0.001, 0.001)	0.006 (−0.006, 0.018)	−0.005 (−0.219, 0.209)	0.014 (0.002, 0.025)	0.005 (−0.208, 0.217)
FFM, kg						
Model 1^b^	0.059 (0.047, 0.071)	0.000 (0.000, 0.001)	0.030 (0.014, 0.045)	0.161 (−0.122, 0.443)	0.019 (0.003, 0.034)	−0.160 (−0.440, 0.121)
Model 2^c^	0.049 (0.039, 0.060)	0.000 (0.000, 0.001)	0.026 (0.010, 0.042)	0.137 (−0.156, 0.431)	0.014 (−0.001, 0.030)	−0.137 (−0.429, 0.155)
Model 3^d^	0.050 (0.039, 0.061)	0.001 (0.000, 0.001)	0.029 (0.014, 0.045)	0.190 (−0.094, 0.475)	0.012 (−0.003, 0.0027)	−0.189 (−0.472, 0.094)

Data are presented as regression coefficient (b, 95% CI)

^a^Associations with nocturnal EE, RER, carbohydrate oxidation and fat oxidation were based on *N*=134

^b^Model 1: adjusted for age and sex, except when the variable was the predictor of interest

^c^Model 2: as for model 1, additionally adjusted for FM (kg) and FFM (kg), except when the variable was the predictor of interest

^d^Model 3: as for model 2, additionally adjusted for HOMA-IR, fasting NEFA and fasting TG, except when the variable was the predictor of interest

### Fasting TG and body composition as predictors of nocturnal EE and substrate oxidation

To compare the strengths of the associations between factors of nocturnal substrate oxidation and EE as presented in Table 2, we provide results of the standardised (*z* score) regression coefficients. As presented in Table 3, the larger regression coefficient for FFM and non-overlapping CIs suggests a significantly stronger association between FFM and absolute nocturnal EE compared with FM. Additionally, FFM showed a stronger association with absolute nocturnal carbohydrate oxidation than fasting TG, although the CIs partially overlap. When nocturnal carbohydrate oxidation was expressed relative to EE, only fasting TG remained significantly associated with nocturnal carbohydrate oxidation. The larger regression coefficient for fasting TG and non-overlapping CIs suggests a significantly stronger inverse association between fasting TG and absolute nocturnal fat oxidation compared with FM. When nocturnal fat oxidation was expressed relative to EE, fasting TG again remained the only factor significantly inversely associated (Table 3).

**Table 3:** Standardised linear regression analyses of significant associations between participant characteristics and nocturnal EE and substrate oxidation

Variable	EE (*z*)^a^	RER (*z*)^a^	Carbohydrate oxidation (*z*)^a^	Carbohydrate oxidation % (*z*)^a^	Fat oxidation (*z*)^a^	Fat oxidation % (*z*)^a^
Fasting TG (*z*)						
Model 1^b^	0.012 (−0.131, 0.156)	**0.336 (0.170, 0.501)**	**0.271 (0.113, 0.428)**	**0.337 (0.171, 0.502)**	**−0.293 (−0.463, −0.123)**	**−0.337 (−0.502, −0.171)**
Model 2^c^	−0.013 (−0.108, 0.082)	**0.338 (0.170, 0.505)**	**0.266 (0.117, 0.416)**	**0.339 (0.171, 0.506)**	**−0.314 (−0.479, −0.149)**	**−0.353 (−0.518, −0.188)**
Model 3^d^	−0.017 (−0.114, 0.079)	**0.337 (0.165, 0.508)**	**0.266 (0.113, 0.419)**	**0.337 (0.166, 0.509)**	**−0.318 (−0.487, −0.149)**	**−0.352 (−0.520, −0.183)**
FM (*z*)						
Model 1^b^	**0.461 (0.338, 0.583)**	0.077 (−0.102, 0.255)	0.213 (0.049, 0.376)	0.078 (−0.101, 0.256)	**0.225 (0.048, 0.402)**	0.078 (−0.101, 0.256)
Model 2^c^	**0.337 (0.237, 0.436)**	0.053 (−0.132, 0.239)	0.141 (−0.023, 0.305)	0.054 (−0.131, 0.240)	**0.180 (−0.002, 0.362)**	0.054 (−0.131, 0.240)
Model 3^d^	**0.320 (0.214, 0.427)**	−0.005 (−0.194, 0.183)	0.089 (−0.079, 0.258)	−0.004 (−0.193, 0.184)	**0.222 (0.036, 0.408)**	−0.004 (−0.193, 0.184)
FFM (*z*)						
Model 1^b^	**0.866 (0.692, 1.040)**	0.160 (−0.121, 0.440)	**0.476 (0.226, 0.726)**	0.160 (−0.121, 0.440)	0.336 (0.058, 0.615)	−0.160 (−0.440, 0.121)
Model 2^c^	**0.724 (0.567, 0.880)**	0.137 (−0.155, 0.429)	**0.416 (0.158, 0.674)**	0.137 (−0.155, 0.428)	0.260 (−0.026, 0.546)	−0.137 (−0.428, 0.155)
Model 3^d^	**0.740 (0.580, 0.900)**	0.190 (−0.093, 0.473)	**0.469 (0.217, 0.721)**	0.189 (−0.094, 0.471)	0.217 (−0.062, 0.496)	−0.189 (−0.471, 0.094)

Data are presented as standardised regression coefficient (β, 95% CI)

All predictors and outcomes are *z*-transformed, and significant associations are shown in bold

^a^Associations with nocturnal RER, fat oxidation, carbohydrate oxidation and EE were based on *N*=134

^b^Model 1: adjusted for age and sex, except when the variable was the predictor of interest

^c^Model 2: as for model 1, additionally adjusted for FM (kg) and FFM (kg), except when the variable was the predictor of interest

^d^Model 3: as for model 2, additionally adjusted for HOMA-IR, fasting NEFA and fasting TG, except when the variable was the predictor of interest

## Discussion

We previously demonstrated that young lean individuals exhibit 24 h rhythmicity in whole-body substrate metabolism, characterised by a higher reliance on carbohydrate oxidation during the day and a shift to fat oxidation at night [4]. This 24 h metabolic flexibility is blunted in older individuals with overweight/obesity and impaired glucose tolerance and insulin sensitivity, who show reduced nocturnal fat oxidation and fail to reach a true fasted state [5]. Since the two populations featured clear differences in participant characteristics, it remained unclear which were associated with this lower nocturnal fat oxidation. Here, we combined data from 18 human clinical trials that included overnight stays in a whole-room metabolic chamber with participants varying in age, body composition and metabolic health. We observed that nocturnal RER and carbohydrate oxidation (absolute and relative to EE) were higher, and fat oxidation (relative to EE) was lower, in older individuals with overweight/obesity with or without type 2 diabetes compared with young and older lean groups. Interestingly, substrate oxidation levels in the older lean population matched the young lean population, suggesting that age per se is not a major factor associated with nocturnal substrate oxidation. Moreover, higher fasting TG levels were most strongly associated with higher nocturnal RER and carbohydrate oxidation (relative to EE) and lower nocturnal fat oxidation (absolute and relative to EE).

Our findings show that older individuals with overweight/obesity with or without type 2 diabetes exhibit higher nocturnal RER and carbohydrate oxidation (absolute and relative to EE) and reduced nocturnal fat oxidation (relative to EE) compared with young and older lean groups. These findings are consistent with our previous studies showing that nocturnal RER did not decrease during the night in older individuals with overweight/obesity and impaired glucose tolerance and insulin sensitivity, as opposed to a decrease in nocturnal RER observed in young lean individuals [4, 5]. Another study also found higher nocturnal carbohydrate oxidation in obese individuals both with and without type 2 diabetes, as compared with normal-weight, active individuals [13]. In all included studies, participants were fed in energy balance and macronutrient intake was similar and controlled across groups, with total daily macronutrient distribution of approximately 50% energy from carbohydrates, 35% energy from fat and 15% energy from proteins. Therefore, the source fuelling the differential carbohydrate oxidation remains speculative. It is well known that individuals with overweight/obesity with or without type 2 diabetes are characterised by increased endogenous glucose production [14–16], predominantly driven by enhanced hepatic gluconeogenesis [17, 18]. Moreover, adipose tissue insulin resistance promotes increased lipolysis and consequently increases the contribution of glycerol as a substrate for gluconeogenesis [17–19]. As such, higher rates of gluconeogenesis are probably attributable to higher levels of nocturnal carbohydrate oxidation in individuals with overweight/obesity with or without type 2 diabetes. Alternatively, the higher glucose oxidation in older overweight/obesity with or without type 2 diabetes groups could be attributable to a reduced capacity to switch to fat oxidation in those individuals. In that context, we have shown that individuals with type 2 diabetes in general are characterised by lower mitochondrial capacity compared with healthy control individuals [1]. Furthermore, the day–night rhythmicity in mitochondrial capacity that is observed in young lean individuals is absent in older individuals with overweight/obesity and impaired glucose tolerance and insulin sensitivity [5]. Exercise training, known to improve mitochondrial function, was, however, not able to restore the impaired nocturnal fed-to-fasting transition in whole-body substrate oxidation in men with insulin resistance [20].

Interestingly, our findings showed that nocturnal EE (adjusted for FFM), RER, and carbohydrate (absolute and relative to EE) and fat oxidation (absolute and relative to EE) were comparable between young and older lean groups, consistent with previous findings [21, 22]. Together, these findings suggest that age per se may not be a major factor in the higher nocturnal RER and carbohydrate oxidation and lower fat oxidation observed in older individuals with overweight/obesity with or without type 2 diabetes. Instead, it is likely that overweight/obesity-related metabolic factors, such as hyperglycaemia or insulin resistance, may drive the lower nocturnal fat oxidation [2]. Nonetheless, our results did not show an association between HOMA-IR and nocturnal substrate oxidation. However, it is crucial to note that we did not have access to 24 h blood samples. As such, we cannot rule out the possibility that elevated nocturnal glucose and insulin levels, commonly observed in individuals with obesity and type 2 diabetes, may still influence nocturnal substrate oxidation.

We found that elevated fasting TG levels were most strongly associated with higher nocturnal RER and carbohydrate oxidation (relative to EE) and lower fat oxidation (absolute and relative to EE), suggesting that they may represent a potential marker of overnight substrate utilisation. This association is independent of diet and probably reflects disturbed TG metabolism and overall substrate oversupply in older individuals with overweight/obesity with or without type 2 diabetes. We previously demonstrated that, compared with young lean individuals, older individuals with overweight/obesity and impaired glucose tolerance and insulin sensitivity exhibited higher 24 h TG levels which remained high for a more prolonged time period (until after midnight) following dinner, suggesting a prolonged fed state compared with lean individuals [5]. Elevated fasting TG levels in individuals with insulin resistance may derive from increased hepatic VLDL-TG production and reduced TG clearance due to lower lipoprotein lipase activity [23, 24]. Moreover, elevated fasting TG levels have been shown to correlate positively with hepatic insulin resistance [25], suggesting that hepatic insulin resistance may represent the underlying determinant, with fasting TG serving as an associated biomarker. However, our cross-sectional study design does not allow us to prove this suggestion directly.

Since it is known that fasting can elicit beneficial metabolic adaptations, inducing a nocturnal fasted state characterised by an increase in fat oxidation and reduction in carbohydrate oxidation, it may be advantageous for improving long-term metabolic health in individuals with overweight/obesity with or without type 2 diabetes. In that context, extending the daily fasting period through time-restricted eating regimens has been shown to stimulate beneficial metabolic effects including increased lipid oxidation [26] and improved insulin sensitivity [27], although results are not consistent and demand further investigation.

Our study has several strengths including the use of whole-room calorimetry, allowing for highly controlled and standardised conditions across all participants (e.g. fed in energy balance, providing similar macronutrient distribution, and controlled physical activity). Additionally, the study design included a comparison of four distinct groups, varying in age, metabolic health status and body composition, providing early insight into the impact of characteristics on nocturnal substrate oxidation. However, some limitations should be noted as well. Group sample sizes were imbalanced: for example, the young and older lean groups included fewer participants compared with the older overweight/obese with or without type 2 diabetes groups. However, this imbalance was not by design but resulted from the availability of participant data from prior studies. Another limitation is that the dataset lacked a continuous age distribution and sex was not evenly distributed across groups, with the young lean group consisting only of men. Due to the nature of the human clinical trials conducted, the timing of the dinner meal prior to entry into the respiration chamber may have varied slightly between groups. However, because nocturnal EE and substrate oxidation were calculated over the entire night, it is unlikely to have contributed to the observed differences in nocturnal substrate oxidation. Nocturnal EE was determined during sleep within a fixed time interval and across three shorter intervals, although potential inter-individual differences in circadian rhythms cannot be fully excluded. Furthermore, we did not have urinary nitrogen measurements to determine protein oxidation, but protein oxidation was estimated as a fixed percentage of EE [12]. Since the analyses were cross-sectional, causal relationships cannot be deduced from our study. Nevertheless, our findings provide a first step towards identifying predictors of nocturnal EE and substrate oxidation. Future studies in larger, more diverse and more extensively phenotyped populations are needed to validate and extend these observations.

In conclusion, older individuals with overweight/obesity with or without type 2 diabetes exhibit higher RER and carbohydrate oxidation (absolute and relative to EE) and lower fat oxidation (relative to EE) during the night, compared with young and older lean individuals. Since young and older lean individuals showed similar substrate oxidation, our findings suggests that age is not a major factor in determining nocturnal substrate oxidation. Fasting TG levels were positively associated with nocturnal RER and carbohydrate oxidation (absolute and relative to EE), and negatively associated with nocturnal fat oxidation (absolute and relative to EE), suggesting a potential role for fasting TG as a metabolic marker of substrate utilisation during the overnight fasting period. Further studies are warranted to investigate the causal relationship and underlying mechanisms between fasting TG and nocturnal substrate oxidation.

## Supplementary Information

Below is the link to the electronic supplementary material.

ESM (PDF 324 KB)

## Data Availability

Datasets of this study can be made available upon reasonable request.

## Abbreviations

AIC: Akaike’s information criterion
DEXA: Dual energy x-ray absorptiometry
DPP-4: Dipeptidyl peptidase-4
EE: Energy expenditure
FFM: Fat-free mass
FM: Fat mass
LMM: Linear mixed model
OBE: Older individuals with overweight/obesity
OL: Older lean individuals
RER: Respiratory exchange ratio
T2D: Older individuals with overweight/obesity and type 2 diabetes
TG: Triglycerides
${\dot{V}\mathrm{CO}}_{2}$: Volume of carbon dioxide
VIF: Variance inflation factor
${\dot{V}\mathrm{O}}_{2}$: Volume of oxygen
YL: Young lean individuals
