# Supplementary material for: Nocturnal fat oxidation is lower in older individuals with overweight/obesity, including those with type 2 diabetes, and is associated with fasting triglyceride levels
Source: Diabetologia. 2026 May 12;69(8):2347–59. doi: 10.1007/s00125-026-06736-z (PMC13310229; doi:10.1007/s00125-026-06736-z)

ESM Table 1. Overview of experimental conditions across included studies

ID	Publication reference	Design	Intervention	Data used (respiration chamber data)	Participants	Standardization prior to overnight indirect calorimetry measurement
NCT02261168	Van Moorsel et al. [1]	Observational study	No intervention	Pre- intervention measurement	12 young lean men	Standardized breakfast was consumed at home (9:00), standardized lunch (14:00) and dinner (19:00) were provided at the research facility
NCT02835664	Remie et al. [2]	Randomized, double-blinded, placebo-controlled crossover intervention	6 weeks nicotinamide riboside supplementation versus placebo	End of placebo arm	13 older men and women with overweight/obesity	Standardized dinner was provided at the research facility (18:30)
NCT03338855	Op den Kamp et al [3]	Randomized, double blinded, placebo-controlled crossover intervention	5 weeks dapagliflozin supplementation versus placebo	End of placebo arm	24 older men and women with overweight/obesity and type 2 diabetes	Standardized breakfast (8:30), lunch (13:00) and dinner (18:00) were provided at the research facility
NCT03593343	Roumans et al. [4]	Randomized cross over trial	16h vs 9.5h fasting	16h fast arm	11 older men and women with overweight/obesity and non-alcoholic fatty liver	Standardized breakfast (8:00), lunch (12:00) and dinner (16:30) were provided at the research facility
NCT04510155	Roumans et al. [4]	Randomized cross over trial	16h vs 9.5h fasting	16h fast arm	10 older lean men and women	Standardized breakfast (8:00), lunch (12:00) and dinner (16:30) were provided at the research facility
NCT03721874	Veelen et al. [5]	Randomized, double blinded, placebo-controlled crossover intervention	Two weeks dapagliflozin supplementation versus placebo	End of placebo arm	14 older men and women with overweight/obesity and prediabetic insulin-resistance	Standardized breakfast (8:00), lunch (12:00), snack (16:00) and dinner (18:00) were provided at the research facility
NCT03733743	Wefers et al. [6]	Observational study	No intervention	Pre- intervention measurement	12 older men with overweight/obesity and prediabetic insulin-resistance	Standardized breakfast (8:00), lunch (13:00) and dinner (18:00) were provided at the research facility
NCT03800290	Van Beek et al. [7]	Randomized, double blinded, placebo-controlled crossover intervention	2 weeks clenbuterol supplementation versus placebo	End of placebo arm	11 young lean men	Standardized dinner (18:00) was provided at the research facility
NCT03992248	Andriessen et al. [8]	Randomized, controlled, crossover trial	3 weeks of time restricted eating: 10h vs 14h eating regime (14h = control condition)	End of control arm	14 older men and women with overweight/obesity and type 2 diabetes.	Standardized breakfast (8:00), lunch (12:00), snack (15:00) and dinner (18:40) were provided at the research facility
NCT02580513	Wefers et al. [9]	Randomized, controlled, crossover trial	Rapid day-night shift protocol versus control	End of control arm	14 young lean men	Standardized breakfast (8:00), lunch (12:30), snack (15:00) and dinner (20:00) were provided at the research facility
NCT03662984	De Wit-Verheggen [10]	Randomized, double blinded, placebo-	35 days of ciprofibrate supplementation versus placebo	End of placebo arm	10 older insulin-resistant men	Standardized dinner (17:00) was provided at the research facility

		controlled crossover intervention				
NCT05263232	Harmsen et al. [11]	Randomized crossover trial	4,5 days of natural light versus artificial light exposure	End of the constant artificial lighting condition (Nocturnal EE/RER did not differ between light conditions)	13 older men and women with overweight/obesity and type 2 diabetes	Standardized breakfast (9:00), lunch (14:00) and dinner (19:00) were provided at the research facility
NCT05463575	Koene et al. (not published)	Randomized, double blinded, placebo-controlled crossover intervention	42 days of ketohexokinase inhibitor supplementation versus placebo	End of placebo arm	14 older men and women with overweight/obesity with non-alcoholic fatty liver disease	Standardized dinner (18:00) was provided at the research facility
NCT05073068	Kotte et al. (not published)	Randomized, controlled, cross-over trial	Acute morning versus evening exercise versus no-exercise	Pre- intervention measurement	18 older men and women with overweight/obesity and prediabetic insulin-resistance	Standardized breakfast (8:00), lunch (12:00), snack (16:00) and dinner (19:00) were provided at the research facility
NCT03829982	Harmsen et al. [12]	Randomized, controlled, crossover trial	Bright light versus dim light exposure	Pre- intervention measurement	14 older men and women with overweight/obesity and prediabetic insulin-resistance	Standardized breakfast (8:00), lunch (13:00) and dinner (18:00) were provided at the research facility
NCT00998504	Timmers et al. [13]	Randomized, double blinded, placebo-controlled crossover intervention	30 days of resveratrol supplementation versus placebo	End of placebo arm	11 older men with obesity	Standardized dinner (18:00) was provided at the research facility
NCT04565418	Harmsen et al. [14]	Single-arm longitudinal design	12 weeks of exercise training	Pre- intervention measurement	10 older men with overweight/obesity and prediabetic insulin-resistance	Standardized breakfast (8:00), lunch (13:00) and dinner (18:00) were provided at the research facility
NTR7426	Vanweert [15]	Randomized, double blinded, placebo-controlled crossover intervention	2 weeks of sodium phenylbutyrate supplementation versus placebo	End of placebo arm	16 older men and women with overweight/obesity and type 2 diabetes	Standardized dinner (18:30) was provided at the research facility

References

[1] van Moorsel D, Hansen J, Havekes B, et al. (2016) Demonstration of a day-night rhythm in human skeletal muscle oxidative capacity. Mol Metab 5(8): 635-645. 10.1016/j.molmet.2016.06.012

[2] Remie CME, Roumans KHM, Moonen MPB, et al. (2020) Nicotinamide riboside supplementation alters body composition and skeletal muscle acetylcarnitine concentrations in healthy obese humans. Am J Clin Nutr 112(2): 413-426. 10.1093/ajcn/nqaa072

[3] Op den Kamp YJM, de Ligt M, Dautzenberg B, et al. (2021) Effects of the SGLT2 Inhibitor Dapagliflozin on Energy Metabolism in Patients With Type 2 Diabetes: A Randomized, Double-Blind Crossover Trial. Diabetes Care 44(6): 1334-1343. 10.2337/dc20-2887

[4] Roumans KHM, Veelen A, Andriessen C, et al. (2023) A prolonged fast improves overnight substrate oxidation without modulating hepatic glycogen in adults with and without nonalcoholic fatty liver: A randomized crossover trial. Obesity (Silver Spring) 31(3): 757-767. 10.1002/oby.23676

[5] Veelen A, Andriessen C, Op den Kamp Y, et al. (2023) Effects of the sodium-glucose cotransporter 2 inhibitor dapagliflozin on substrate metabolism in prediabetic insulin resistant individuals: A randomized, double-blind crossover trial. Metabolism 140: 155396. 10.1016/j.metabol.2022.155396

[6] Wefers J, Connell NJ, Fealy CE, et al. (2020) Day-night rhythm of skeletal muscle metabolism is disturbed in older, metabolically compromised individuals. Mol Metab 41: 101050. 10.1016/j.molmet.2020.101050

[7] van Beek SMM, Bruls YMH, Vanweert F, et al. (2023) Effect of β2-agonist treatment on insulin-stimulated peripheral glucose disposal in healthy men in a randomised placebo-controlled trial. Nat Commun 14(1): 173. 10.1038/s41467-023-35798-5

[8] Andriessen C, Fealy CE, Veelen A, et al. (2022) Three weeks of time-restricted eating improves glucose homeostasis in adults with type 2 diabetes but does not improve insulin sensitivity: a randomised crossover trial. Diabetologia 65(10): 1710-1720. 10.1007/s00125-022-05752-z

[9] Wefers J, van Moorsel D, Hansen J, et al. (2018) Circadian misalignment induces fatty acid metabolism gene profiles and compromises insulin sensitivity in human skeletal muscle. Proc Natl Acad Sci U S A 115(30): 7789-7794. 10.1073/pnas.1722295115

[10] de Wit-Verheggen VHW, Vanweert F, Raiko J, et al. (2023) The tissue-specific metabolic effects of the PPARα agonist ciprofibrate in insulin-resistant male individuals: a double-blind, randomized, placebo-controlled crossover study. Obesity (Silver Spring) 31(10): 2493-2504. 10.1002/oby.23874

[11] Harmsen J-F, Habets I, Biancolin AD, et al. (2026) Natural daylight during office hours improves glucose control and whole-body substrate metabolism. Cell Metabolism 38(1): 65-81.e10. 10.1016/j.cmet.2025.11.006
[12] Harmsen JF, Wefers J, Doligkeit D, et al. (2022) The influence of bright and dim light on substrate metabolism, energy expenditure and thermoregulation in insulin-resistant individuals depends on time of day. Diabetologia 65(4): 721-732. 10.1007/s00125-021-05643-9
[13] Timmers S, Konings E, Bilet L, et al. (2011) Calorie restriction-like effects of 30 days of resveratrol supplementation on energy metabolism and metabolic profile in obese humans. Cell Metab 14(5): 612-622. 10.1016/j.cmet.2011.10.002
[14] Harmsen JF, Kotte M, Habets I, et al. (2024) Exercise training modifies skeletal muscle clock gene expression but not 24-hour rhythmicity in substrate metabolism of men with insulin resistance. J Physiol 602(23): 6417-6433. 10.1113/jp285523
[15] Vanweert F, Neinast M, Tapia EE, et al. (2022) A randomized placebo-controlled clinical trial for pharmacological activation of BCAA catabolism in patients with type 2 diabetes. Nature Communications 13(1): 3508. 10.1038/s41467-022-31249-9

ESM Table 2. Nocturnal EE, RER and substrate oxidation across YL, OL, OBE and T2D groups

	YL	OL	OBE	T2D	*p*-value (Group)
**EE, kJ/min**	4.90 (4.55-5.35)	4.26 (3.59-4.63)	5.02 (4.55-5.54)	4.95 (4.55-5.36)	0.008
n	37	10	92	48	
**EE, Unstandardised residuals**	4.58 (4.37-4.86)	4.62 (4.24-4.77)	5.01 (4.70-5.26)	5.09 (4.69-5.32)	0.001
n	11	10	92	47	
**RER, VCO_2/VO_2**	0.81 (0.79-0.82)	0.80 (0.79-0.81)	0.83 (0.81-0.85)	0.83 (0.81-0.84)	<0.001
n	37	10	92	48	
**Carbohydrate oxidation, kJ/min**	1.42 (1.18-1.73)	1.15 (0.88-1.20)	1.81(1.40-2.30)	1.76(1.47-2.01)	<0.001
n	37	10	92	48	
**Carbohydrate oxidation, relative to energy expenditure as %**	28.99 (24.30-34.15)	26.72 (23.01-27.78)	35.70 (28.88-44.29)	35.49 (28.28-40.96)	< 0.001
n	37	10	92	48	
**Fat oxidation, kJ/min**	2.62 (2.42-2.88)	2.33 (2.06-2.83)	2.23 (1.87-2.63)	2.31 (1.97-2.62)	0.014
n	37	10	92	48	
**Fat oxidation, relative to energy expenditure as %**	52.95 (47.82-57.61)	55.21 (54.15-58.89)	46.28(37.74-53.05)	46.48 (41.05-53.65)	<0.001
n	37	10	92	48	

Data are presented as median with 25th-75th percentile

Group size: YL: *n*=37; OL: *n*=10; OBE: *n*=92; and T2D: *n*=48

Sample sizes for EE (Unstandardised residuals) is lower due to missing body composition data

Differences are considered significant at $p<0.05$

ESM Table 3. Nocturnal EE, RER and substrate oxidation over the course of the night across YL, OL, OBE and T2D groups

	YL			OL			OBE			T2D			*p*-value (Group)	*p*-value (Time)	*p*-value (Group x Time)
	1[a]	2[b]	3[c]	1[a]	2[b]	3[c]	1[a]	2[b]	3[c]	1[a]	2[b]	3[c]			
**EE, kJ/min**	5.03 (0.12)	4.85 (0.12)	4.85 (0.12)	4.17 (0.23)	4.19 (0.23)	4.23 (0.23)	5.17 (0.08)	5.05 (0.08)	5.06 (0.08)	5.08 (0.11)	4.91 (0.11)	5.03 (0.11)	0.002	0.005	0.305
n	37	37	37	10	10	10	92	92	92	48	48	48			
**RER, VCO_2/VO_2**	0.81 (0.006)	0.81 (0.006)	0.81 (0.006)	0.80 (0.01)	0.81 (0.01)	0.80 (0.01)	0.83 (0.004)	0.84 (0.004)	0.84 (0.004)	0.83 (0.006)	0.83 (0.006)	0.82 (0.006)	< 0.001	0.754	0.022
n	37	37	37	10	10	10	92	92	92	48	48	48			
**Carbohydrate oxidation, kJ/min**	1.51 (0.11)	1.37 (0.11)	1.43 (0.11)	1.09 (0.21)	1.15 (0.21)	1.09 (0.21)	1.86 (0.07)	1.97 (0.07)	1.93 (0.07)	1.82 (0.10)	1.72 (0.10)	1.71 (0.10)	< 0.001	0.843	0.080
n	37	37	37	10	10	10	92	92	92	48	48	48			
**Carbohydrate oxidation, relative to energy expenditure as %**	29.81 (1.86)	28.43 (1.86)	29.48 (1.86)	26.82 (3.58)	27.68 (3.58)	26.98 (3.58)	35.31 (1.18)	38.74 (1.18)	37.82 (1.18)	35.89 (1.63)	35.27 (1.63)	34.11 (1.63)	< 0.001	0.751	0.021
n	37	37	37	10	10	10	92	92	92	48	48	48			
**Fat oxidation, kJ/min**	2.61 (0.72)	2.60 (0.72)	2.55 (0.72)	2.32 (0.74)	2.28 (0.74)	2.38 (0.74)	2.38 (0.72)	2.17 (0.72)	2.21 (0.72)	2.35 (0.72)	2.30 (0.72)	2.42 (0.72)	0.039	0.170	0.067
n	37	37	37	10	10	10	92	92	92	52	52	52			
**Fat oxidation, relative to energy expenditure as %**	52.13 (4.52)	53.51 (4.52)	52.47 (4.52)	55.11 (5.45)	54.25 (5.45)	54.94 (5.45)	46.67 (4.29)	43.26 (4.29)	44.17 (4.29)	46.09 (4.44)	46.70 (4.44)	47.86 (4.44)	< 0.001	0.751	0.021
n	37	37	37	10	10	10	92	92	92	48	48	48			

Data are presented as mean with SEM

Group size: YL: *n*=37; OL: *n*=10; OBE: *n*=92; and T2D: *n*=48

1[a]: night period between 0:30 to 2:00 hours

2[b]: night period between 2:00 to 3:30 hours

3[c]: night period between 3:30 to 5:00 hours

Differences are considered significant at $p<0.05$

ESM Fig. 1. Flowchart of participants included in the analysis

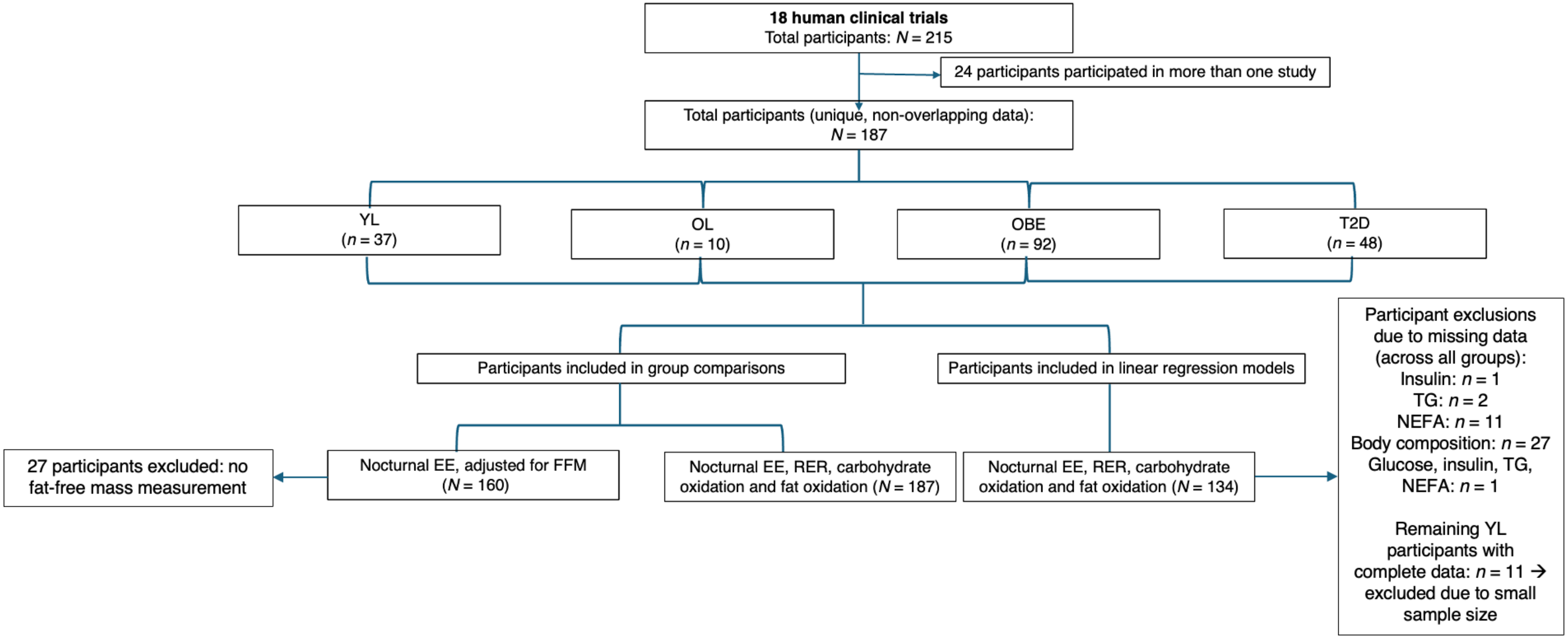

Supplement: Supplementary file 1 — ESM (PDF 324 KB) [file 125_2026_6736_MOESM1_ESM.pdf]
